# Non-Invasive Biomarkers in the Era of Big Data and Machine Learning

**DOI:** 10.3390/s25051396

**Published:** 2025-02-25

**Authors:** Konstantinos Lazaros, Styliani Adam, Marios G. Krokidis, Themis Exarchos, Panagiotis Vlamos, Aristidis G. Vrahatis

**Affiliations:** Bioinformatics and Human Electrophysiology Laboratory, Department of Informatics, Ionian University, 49100 Corfu, Greece

**Keywords:** non-invasive approaches, big data, diagnostics, biomarkers, machine learning

## Abstract

Invasive diagnostic techniques, while offering critical insights into disease pathophysiology, are often limited by high costs, procedural risks, and patient discomfort. Non-invasive biomarkers represent a transformative alternative, providing diagnostic precision through accessible biological samples or physiological data, including blood, saliva, breath, and wearable health metrics. They encompass molecular and imaging approaches, revealing genetic, epigenetic, and metabolic alterations associated with disease states. Furthermore, advances in breathomics and gut microbiome profiling further expand their diagnostic scope. Even with their strengths in terms of safety, cost-effectiveness, and accessibility, non-invasive biomarkers face challenges in achieving monitoring sensitivity and specificity comparable to traditional clinical approaches. Computational advancements, particularly in artificial intelligence and machine learning, are addressing these limitations by uncovering complex patterns in multi-modal datasets, enhancing diagnostic accuracy and facilitating personalized medicine. The present review integrates recent innovations, examines their clinical applications, highlights their limitations and provides a concise overview of the evolving role of non-invasive biomarkers in precision diagnostics, positioning them as a compelling choice for large-scale healthcare applications.

## 1. Introduction

Invasive diagnostic techniques have consistently been vital in assessing and managing a wide range of diseases, providing valuable insights into the underlying pathophysiological processes. The use of biopsies, endoscopies and surgical explorations ensures precise diagnosis and significantly informs clinical decision-making. However, these methods are often associated with substantial disadvantages, including high costs, procedural risks, significant patient discomfort, and logistical complexities in healthcare systems [1,2]. These challenges not only discourage patients from timely diagnostic evaluations but also burden healthcare systems, especially in resource-constrained settings, delaying the initiation of necessary treatments. This situation highlights the pressing need for alternative diagnostic strategies that emphasize accessibility, patient comfort, and cost-effectiveness while maintaining high diagnostic standards. In recent years, non-invasive biomarkers have gained significant attention as a viable alternative, offering the potential to circumvent the drawbacks of invasive procedures. These biomarkers are measurable indicators derived from easily obtainable biological samples or physiological data, such as blood, urine, saliva, stool, sweat, breath, and digital health metrics like heart rate variability [3,4,5]. By providing insights into molecular, cellular, and systemic processes without the need for physically intrusive interventions, non-invasive biomarkers represent a transformative shift in diagnostic methodologies. Non-invasive biomarker methodologies encompass a diverse range of approaches, each offering unique insights into health and disease states. Molecular biomarkers, such as circulating tumor DNA (ctDNA) and microRNAs, derived from blood and other bodily fluids without interventional procedure, reveal genetic mutations, epigenetic changes, and transcriptional anomalies. Proteomic biomarkers, which include proteins and peptides, help uncover disease pathways and inflammatory responses. Metabolomic biomarkers, focusing on small molecules and metabolites, provide a dynamic view of metabolic shifts linked to conditions like cancer, cardiovascular diseases, and diabetes [6,7]. Beyond molecular biomarkers, imaging techniques, such as MRIs, X-rays, and CT scans, play a pivotal role in identifying structural and functional changes in the body [8,9,10].

In recent years, significant progress has been made in non-invasive biomarker detection through advancements in fields such as magnetoresistance-based and graphene-based biosensor technologies, among others. MR biosensors have expanded their applications to include the detection of magnetic nanoparticles, proteins, and DNA, and even the mapping of cardiovascular and brain signals by refining sensor geometries, optimizing surface modifications, and integrating magnetic flux concentrators and microfluidic channels. At the same time, graphene-based biosensors have leveraged graphene’s high specific surface area and exceptional electronic properties to quantitatively detect cancer-related biomarkers such as DNA, miRNA, small molecules, and proteins using diverse signal output techniques like fluorescence, electrochemistry, surface plasmon resonance, and surface-enhanced Raman scattering [11,12].

The present review aims to present a holistic examination of the latest advantages in diverse clinical conditions along with well-established computational methods, including machine learning techniques and tools that are recurrently applied, shedding light on the analytical frameworks underpinning non-invasive biomarker examination. By examining technological breakthroughs and clinical applications, this work attempts to highlight the transformative effects of non-invasive biomarkers, identify their limitations and propose future research directions.

## 2. The Non-Invasive Perspective in Diagnostics and Data Analysis

A comprehensive exploration of non-invasive approaches and related in silico pipelines for data analysis is provided. Blood samples, though considered minimally invasive, remain critical in this direction. They enable comprehensive profiling of intracellular and peripheral biomarkers, providing invaluable information for disease monitoring and early diagnosis [13,14]. Clinical samples like urine, saliva, and sweat are vital for non-invasive assessments, revealing biochemical and hormonal changes. Wearable devices that monitor metrics like the heart rate, respiratory rate, and physical activity, alongside clinical and demographic data, offer a continuous, personalized assessment of physiological states. Collectively, these methodologies highlight the multifaceted nature of non-invasive biomarker discovery, indicating molecular insights with physiological and systemic alterations for a holistic understanding of health. Recent studies on innovative approaches, including breathomics and gut microbiome profiling, have further expanded the diagnostic possibilities [15,16,17,18]. Breathomics analyzes the volatile organic compounds in exhaled breath and shows promise in detecting respiratory diseases and other systemic conditions. Meanwhile, gut microbiome profiling examines the microbial diversity and composition in stool samples, relating it to a range of disorders, ranging from gastrointestinal to neuropsychiatric conditions. These breakthroughs have brought new perspectives to diagnostics, targeting previously underexplored aspects of human health.

Non-invasive biomarkers facilitate more frequent and convenient testing, essential for managing chronic conditions and conducting population health screenings. The reduced procedural risks enhance patient safety and compliance, particularly for vulnerable groups such as children, the elderly, and individuals with multiple health issues [19,20,21]. Moreover, the lower cost and infrastructure demands of non-invasive diagnostics make them an appealing option for large-scale healthcare applications, especially in resource-limited settings. By enabling earlier detection and intervention, non-invasive approaches have the potential to improve clinical outcomes while reducing long-term healthcare expenditures. Nevertheless, despite their promise, non-invasive biomarkers currently face challenges in achieving diagnostic sensitivity and specificity comparable to invasive techniques, limiting their integration into routine clinical practice. Tackling this gap is crucial to fully harnessing their potential in precision medicine.

**Figure 1:**
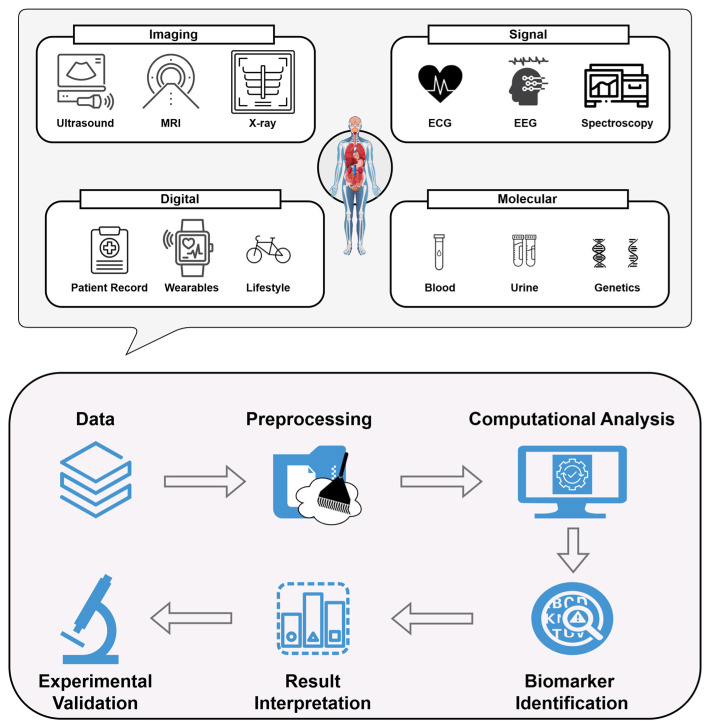
Framework for the identification of various types of non-invasive biomarkers, showcasing data integration derived from imaging analyses, signal processing, digital records and molecular techniques (upper). A graphical illustration of the key steps typically undertaken during computational-based analyses of these data (lower). The workflow encompasses data acquisition, preprocessing, and computational analysis, followed by biomarker identification and interpretation. Experimental validation is conducted to confirm the reliability of the detected markers, reflecting the iterative nature of translating multi-source data into clinically insightful knowledge.

Recent advances in computational technologies are driving significant progress in clinics from a non-invasive perspective. The integration of artificial intelligence (AI), machine learning (ML), and high-throughput data analysis has enabled researchers to uncover complex patterns and predictive markers within large, multi-modal datasets spanning genomics, proteomics, metabolomics, and medical imaging [22,23,24]. These tools enhance the diagnostic accuracy of non-invasive biomarkers and facilitate the stratification of patients into clinically relevant subgroups, paving the way for personalized therapeutic strategies. Such progress is especially valuable for complex, multifactorial diseases like cancer, cardiovascular disorders, and neurodegenerative conditions, where early and accurate diagnosis is crucial. While computational advances have bolstered the potential of non-invasive biomarkers, computational challenges still remain. Translating AI-enhanced biomarker discoveries into clinical practice requires rigorous validation, standardized methodologies, and strong regulatory frameworks to ensure reliability and reproducibility [25]. Additionally, the limited access to advanced computational resources and diverse datasets poses a risk of further exacerbating disparities in healthcare [25,26,27,28]. In light of this, Figure 1 presents different types of non-invasive biomarkers and the principal stages of computational analysis. In more detail, the upper part of the figure highlights commonly utilized non-invasive techniques for biomarker identification across a broad spectrum of diseases and conditions, while the lower one delineates the sequential steps typically followed during computational analyses.

## 3. Recent Advances in Non-Invasive Biomarker Identification

Numerous studies have explored the role of non-invasive biomarkers in specific diseases. The aim of the present study is intentionally broad, encompassing a range of conditions and diseases instead of focusing on a single pathology. By adopting this generalized approach, this work focuses on the identification and assessment of methodologies that span diverse applications, offering a holistic perspective on the landscape of the non-invasive biomarker field. The selected time frame for the current review encompasses studies published between 2019 and 2024, reflecting the latest advancements and emergent trends in this rapidly evolving field. Table 1 outlines the prevalent data types and formats employed in relevant studies, offering insight into the modalities in which biomarker information is captured. The methods are categorized according to the data types detailed in Table 1, resulting in four primary subdivisions: imaging-based, molecular-based, signal-based, and clinical-based studies.

### 3.1. Imaging-Data-Based Studies

Keinz et al. [29] demonstrated how imaging data can enable non-invasive biomarker identification for diagnosing deep vein thrombosis (DVT). Using compression ultrasound imaging from 255 volunteers and validating it on 83 patients, their study focused solely on imaging, avoiding genomic techniques. They developed AutoDVT, a CNN-based segmentation model to assess vein compressibility, enabling non-specialists to diagnose DVT at the point of care. Separate models were used for different anatomical regions, incorporating real-time feedback for image acquisition. The system achieved sensitivity between 82 and 96% and specificity from 70 to 82%, showing strong diagnostic potential. Despite the promising results, the limitations included a small sample size and the need for larger multi-center trials.

Ottakath et al. [30] explored non-invasive biomarker identification for carotid artery stenosis using automated ultrasound image segmentation. Analyzing 971 B-mode ultrasound images from Toshiba (Tokyo, Japan) and Ultrasonix (Birmingham, UK) devices, the study leveraged the bi-attention DoubleUNet architecture, integrating channel-wise and spatial attention. Images were resized to 224 × 224 pixels and split into training, validation, and test sets. The model achieved state-of-the-art performance, with a 97.92% Dice coefficient, 95.96% IoU, 98.35% precision, and 97.57% recall, surpassing traditional segmentation methods. Guided back-propagation and attention maps enhanced the interpretability. While the results demonstrate high accuracy, future work aims to improve the dataset quality and the expand clinical applicability.

Furthermore, Villanueva et al. [31] developed a non-invasive method for assessing portal hypertension using Doppler ultrasound-derived hepatic blood flow waveforms and computational modeling. Data from the hepatic vein (HV), portal vein (PV), and hepatic artery (HA) were collected from two patients—one with a normal and one with an elevated hepatic venous pressure gradient (HVPG). A 0D computational cardiovascular model simulated the blood flow and pressure distributions, with CMA-ES optimizing the patient-specific parameters. The model accurately reproduced the normal and altered hepatic waveforms, closely matching the invasive measurements. Sensitivity analysis highlighted liver compliance and vascular resistance as key determinants of the HVPG, demonstrating the model’s potential for non-invasive portal pressure estimation.

Moreover, Sun et al. [32] developed a non-invasive method for diagnosing pancreatic steatosis (PS) using ultrasound imaging and deep learning. Analyzing grayscale ultrasound images from 139 patients, the study employed an AlexNet CNN fine-tuned on ImageNet for PS classification. Data augmentation improved the model robustness, and images were resized to 512 × 512 pixels for training. The model outperformed traditional radiological assessments, achieving an AUC of 0.901, 89.5% sensitivity, and 81.4% accuracy in training, with validation results of AUC 0.837, 92.0% sensitivity, and 85.7% accuracy. Sun et al. advocated for its use in early screening and T2DM risk assessment, although further validation is needed.

Furthermore, Keskenler et al. [23] developed a non-invasive skin cancer diagnosis method using dermoscopic image analysis from the HAM10000 dataset (10,015 high-resolution images of eight skin lesion types). Biomarkers were extracted from the luminance, RGB values, and texture patterns, combined with clinical data (age, gender, lesion location). A multi-layer machine learning (MLML) architecture processed the images in three stages: (1) initial classification using decision tree, random forest, neural network, naïve Bayes, and SVM, (2) refinement with K-nearest neighbor (KNN), and (3) accuracy enhancement via linear regression. The model achieved 88.81% accuracy, 88.89% precision, 99.17% recall, and a 93.75% F1-score, demonstrating its potential for non-invasive skin cancer diagnosis.

In a similar vein, Alfi et al. [33] developed a non-invasive diagnostic approach for melanoma, integrating deep learning (DL) with ensemble stacking of machine learning (ML) models to improve lesion classification. Using 3297 dermoscopic images from the ISIC 2018 dataset, the study applied preprocessing techniques like normalization and augmentation. A hybrid method combined classical ML models (SVM, random forest, logistic regression, KNN, gradient boosting) with pre-trained CNNs (MobileNet, Xception, ResNet50, ResNet50V2, DenseNet121) via transfer learning. The best-performing ensemble model achieved 92% accuracy and an AUC of 0.97. SHAP heatmaps were used to enhance the interpretability, highlighting key features indicative of melanoma.

Moreover, Qu et al. [34] explored non-invasive thermal imaging biomarkers for diagnosing and monitoring pneumonia, including COVID-19 cases. By analyzing the back temperature patterns, the study identified correlations with respiratory conditions, providing a practical tool for early diagnosis. Data from 69 subjects, including pneumonia patients and healthy controls, were collected using a smartphone-connected thermal imager. A computational pipeline involving preprocessing, feature extraction, and classification was implemented, with PCA reducing the dimensionality. Among the tested ML models (SVM, KNN, decision trees, LDA, QDA), SVM achieved the highest accuracy—93% for binary classification and 81% for ternary classification. The system effectively tracked disease progression, although further validation with larger cohorts is needed before clinical adoption.

In addition, Maddali et al. [64] investigated non-invasive biomarker identification for idiopathic pulmonary fibrosis (IPF) using deep learning on CT scans. By analyzing the radiologic patterns with a 3D ResNet-based CNN ensemble, the study aimed to replace invasive lung biopsies. Data from over 2000 patients with interstitial lung disease (ILD) were processed using augmentation techniques and hyperparameter tuning to enhance the model robustness. The model achieved an AUC of 0.87, with 67% sensitivity and 90% specificity, outperforming clinician assessments. External validation confirmed its consistency across CT scanners. While promising, the study’s retrospective design necessitates further prospective validation before clinical adoption.

In the same vein, Ye et al. [65] identified non-invasive imaging biomarkers from multimodal CT scans to predict the pathological complete response (pCR) in non-small-cell lung cancer (NSCLC) patients undergoing neoadjuvant immunochemotherapy. Analyzing CT scans from 225 patients, the study used deep learning to extract imaging features, integrating non-contrast and contrast-enhanced modalities. A foundation model (FM-LCT) trained on lung cancer datasets was employed, with PCA reducing the dimensionality before classification using random forest models. The fused-feature model, LUNAI-fCT, achieved the highest performance (AUC 0.866, 80% accuracy, 91.7% sensitivity, 73.9% specificity). SHAP and Grad-CAM analyses highlighted key tumor regions influencing the predictions. While the model shows potential for personalized cancer care, larger-scale validation is needed to confirm its clinical applicability.

Likewise, Tamehisa et al. [37] developed a non-invasive method for classifying uterine leiomyoma subtypes using MRI-derived quantitative imaging biomarkers to detect MED12 mutations. A computational pipeline extracted signal intensities reflecting collagen and water content, identifying key differences between the subtypes. Support vector classification (SVC) and logistic regression (LR) models were trained and validated using k-fold cross-validation and external datasets. The models demonstrated exceptional performance, with AUC values of 0.974 (SVC) and 0.988 (LR) internally, and perfect AUC and accuracy (1.0) in external validation, highlighting their potential for genetic characterization without invasive procedures.

Van der Kolk et al. [38] investigated radiomics-based features from conventional MRI as non-invasive biomarkers for diagnosing Menière’s disease. Using T2-weighted MRI scans from 120 patients and 140 controls across four medical centers, the study applied machine learning to distinguish between groups. A computational pipeline included manual labyrinth segmentation, voxel normalization, and radiomic feature extraction, reducing 812 features to 15 via PCA. A multi-layer perceptron (MLP) classifier achieved 82% accuracy, 83% sensitivity, 82% specificity, and an AUC of 87%. The model showed strong diagnostic potential but required further validation on larger datasets and automation of the segmentation to enhance the scalability.

Additionally, Rehman et al. [22] developed a non-invasive diagnostic framework for obstructive lung diseases (OLDs) by combining iris imaging and physiological features. The data from 529 subjects included infrared-captured iris images and physiological measurements such as the BMI and blood pressure. A multi-step pipeline involved iris image preprocessing using localization, segmentation, and Dougman’s rubber sheet model, followed by feature extraction with GLCM and GLRL, resulting in 112 features. Feature selection through t-tests and PCA identified key biomarkers. Among the ten machine learning models tested, ensemble learning achieved the highest accuracy (97.6%), followed by SVM (95.6%). The study demonstrated that integrating iris and physiological features improved the diagnostic accuracy compared to individual feature analysis.

Similarly, Wulandari et al. [66] developed a non-invasive anemia detection method using conjunctival imaging, eliminating the need for blood sampling. The “Eyes-defy-anemia” dataset provided segmented conjunctival images for deep learning analysis. A computational pipeline included preprocessing, augmentation, and class balancing with SMOTE and Tomek Links. MobileNetV2 was used for feature extraction, with three classification approaches tested: SVM, MobileNetV2, and a hybrid MobileNetV2-SVM model. The hybrid model achieved the best performance, with 93% accuracy, 91% sensitivity, and 94% specificity, demonstrating the potential of ocular imaging for anemia diagnosis.

Furthermore, Abdeltawabet et al. [39] developed AI-driven non-invasive diagnostic models across multiple medical imaging modalities. Using diffusion-weighted MRI (DWI), cine MRI, and histopathological images, the study applied deep learning to detect renal transplant rejection, analyze cardiac function, and classify kidney cancer. The customized computational pipelines included CNNs for DWI-based rejection detection, FCNs for cardiac MRI segmentation, and pyramidal deep learning frameworks for kidney cancer classification. Preprocessing steps, such as histogram equalization and segmentation, improved the image quality. The renal rejection model achieved 92% accuracy, the cardiac analysis aligned with expert evaluations, and the pyramidal framework outperformed state-of-the-art kidney cancer classifiers. The study underscores deep learning’s potential for broad clinical adoption in non-invasive diagnostics.

In a similar light, Prince et al. [35] developed an explainable AI (XAI) model for non-invasive CNS tumor diagnosis, reducing reliance on biopsies. MRI and CT scans from 50 patients (46,879 2D DICOM images) were preprocessed and resized for deep learning analysis. A ResNet V2-50 model, pretrained on ImageNet, extracted the vector embeddings, which were mapped into a 256-dimensional latent space using an autoencoder and clustered via Gaussian mixture models. The study emphasized user-centered design (UCD), integrating clinician feedback through an iterative process with SHAP saliency maps and Google’s What-If Tool (WIT) to enhance the interpretability. While the final accuracy metrics were not disclosed, the XAI prototype showed strong potential for real-world clinical application in non-invasive CNS tumor diagnosis.

Similarly, Sathyaseelan et al. [67] developed a non-invasive method for detecting strep throat infections using smartphone-captured throat images, replacing traditional swab tests. A smartphone camera with an add-on device enhanced the image quality, ensuring clear visualization. The study employed a multi-task cascaded convolutional neural network (MTCNN) to detect and isolate the throat region, extracting the color, texture, and structural patterns indicative of infection. A binary classification model combining neural networks and decision trees distinguished between infected and healthy cases. The model achieved 95% accuracy, 96% sensitivity, 92% specificity, and an AUC-ROC of 0.97, demonstrating the potential of smartphone-based diagnostics for clinical and remote healthcare applications.

**Figure 2:**
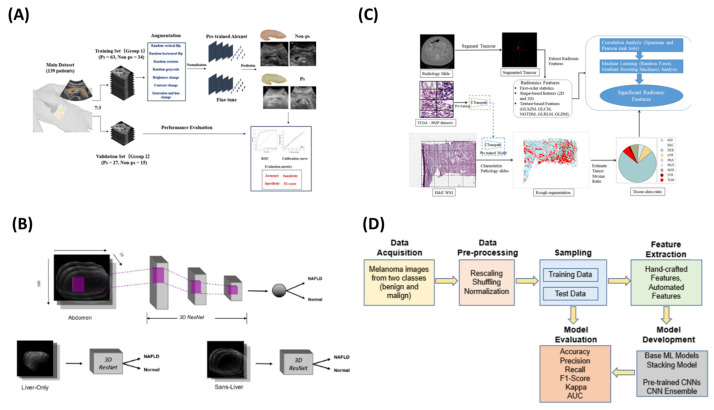
Collage of four representative image-based ML methodologies analyzed in this study. (**A**) showcases the method proposed by Sun et al. [32], **(B**) presents the approach described by Alfi et al. [33], (**C**) depicts the methodology outlined by Adjei et al. [36] and (**D**) illustrates the methodological framework introduced by Pillai et al. [46].

Moreover, Adjei et al. [36] explored non-invasive biomarker identification for colorectal cancer (CRC) using radiomic features from CT scans to complement or replace traditional tumor–stroma ratio (TSR) measurements. The data from the Cancer Genomic Atlas (TCGA) included 459 pathology slides and 451 corresponding CT scans. The radiomic feature extraction followed the IBSI guidelines, while a vision transformer (ViT) model segmented the tumor and stroma areas on the histopathology slides to calculate the TSR. The Spearman’s and Pearson’s correlations identified associations between the radiomic features and the TSR, with random forest (RF) and gradient boosting machine (GBM) ranking the feature importance. Key radiomic features, such as GLSZM_LALGLE, showed strong correlations with the TSR, demonstrating radiomics’ potential as a non-invasive alternative for CRC diagnosis. Figure 2 summarizes the above studies.

Similarly, Pillai et al. [46] developed a non-invasive method for diagnosing non-alcoholic fatty liver disease (NAFLD) using abdominal MRI-derived metrics from the UK Biobank. Key biomarkers, including the proton density fat fraction (PDFF), visceral adipose tissue (VAT), and abdominal subcutaneous adipose tissue (ASAT), were extracted from multi-echo spoiled-gradient-echo MRI scans. A 3D ResNet-based CNN classified NAFLD using the PDFF, while a 2D U-Net segmented the liver for focused analysis. Multi-task learning (MMoE) predicted the PDFF, VAT, and ASAT, achieving up to a 0.95 Spearman’s correlation. The non-local ResNet performed best, with 0.88 precision and a 0.89 F1-score, supporting whole-abdomen MRI as a superior approach to liver-only segmentation for NAFLD assessment.

Last but not least, Chen et al. [40] used radiomics features from post-contrast T1-weighted MRI (T1C) scans to classify meningioma grades, reducing the need for invasive biopsies. MRI data from 150 patients were analyzed, with radiomics features extracted using LIFEx software to assess the tumor aggressiveness. Feature selection involved distance correlation, LASSO, and gradient boosting decision trees (GBDTs), refining the dataset for classification. Machine learning models, including LDA and SVM, were tested, with LDA + LASSO achieving the highest accuracy (75.6%) and a Kappa value of 0.603, outperforming the SVM models. The study reinforces radiomics as a valuable tool for non-invasive tumor grading, although further validation is needed for clinical adoption.

### 3.2. Molecular-Data-Based Studies

Lorenzovici et al. [43] developed a non-invasive computer-aided diagnostic (CAD) system for colorectal cancer (CRC) diagnosis using blood and urine biomarkers. A dataset of 33 clinical biomarkers, including albumin, bilirubin, and creatinine, was analyzed alongside qualitative factors like the tumor position and living environment. A machine learning pipeline in MATLAB (MATLAB, 2020, The MathWorks Inc.: Natick, MS, USA) applied binary classification models and artificial neural networks (ANNs) for regression tasks. The deep neural network achieved the most precise predictions, with the linear discriminant analysis reaching 77.8% accuracy and the regression models yielding a mean squared error of 0.0000529. The study highlights the potential of combining fluid biomarkers with AI to improve CRC diagnostics through non-invasive early detection. Table 2 provides an overview of conditions frequently analyzed, revealing the wide range of applications in this field.

Moreover, Javanshir et al. [47] identified biomarkers distinguishing non-invasive and invasive pancreatic cancer, emphasizing early detection for improved prognosis. Using microarray datasets (GSE62165, GSE19281) from the GEO database, the study performed differential expression analysis (GEO2R) and intersected DEGs via Venn diagrams. Functional annotation was conducted through KEGG and Gene Ontology (GO) analyses, while PPI networks (STRING, Cytoscape) identified hub genes. Validation using TCGA data confirmed eight key proteins (SPARC, THBS2, COL11A1, COL1A1, COL1A2, COL3A1, SERPINH1, PLAU) linked to cell adhesion and ECM regulation, with high expression correlating with increased mortality, highlighting their role in pancreatic cancer progression.

In a similar vein, Demir et al. [44] developed an AI-driven non-invasive diagnostic method for bladder cancer by analyzing the droplet patterns from blood and urine samples. Using light microscopy and a ResNet-18 convolutional neural network (CNN), the study extracted the morphological features of dried droplets to classify samples as “bladder cancer” or “non-cancerous”. Transfer learning refined the model, which was validated through five-fold cross-validation. The AI-assisted method achieved 97.3% accuracy, 97.7% sensitivity, and 97.2% specificity for blood samples, while urine samples analyzed with KCl solution reached 95.3% accuracy, 98.7% sensitivity, and 82.9% specificity. The study highlights AI’s potential in transforming non-invasive cancer diagnostics.

Additionally, Sajid et al. [45] developed a non-invasive method for coronary artery disease (CAD) diagnosis and risk stratification using clinical, chemical, and molecular biomarkers combined with machine learning (ML). Data from 36 pre-angiography biomarkers, including miRNA33a and miRNA146a, were processed through a multi-stage computational pipeline with feature selection identifying the top 15 biomarkers. Random forest, gradient boosting, and extreme gradient boosting models predicted the Gensini scores, stenosis percentage, and vessel involvement, achieving 86.26% accuracy for the Gensini group prediction, 90.91% for the CAD severity stratification, and 82.58% for the vessel involvement. Regression tasks yielded an R-squared value of 0.58 for the stenosis percentage prediction. The AI-CADR risk stratification framework demonstrated strong clinical decision-making potential, providing a cost-effective, non-invasive alternative to coronary angiography.

**Figure 3:**
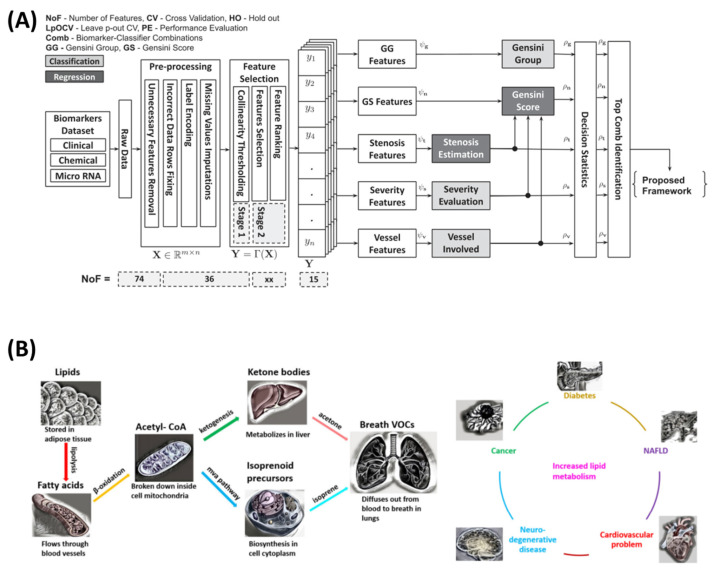
Collage of two indicative molecular-based non-invasive methodologies. (**A**) Methodology presented by Sajid et al. for risk stratification [45]. (**B**) Schematic illustrating the origin of breath VOCs from lipid metabolism and their association with conditions characterized by high lipid metabolic rates from the study by Souradeep et al. [74].

Moreover, Souradeep et al. [74] developed a non-invasive VOC analysis framework using resistive sensing technology and machine learning to detect acetone and isoprene in exhaled breath, aiding disease diagnosis related to lipid metabolism. Data were collected from an indium-oxide-based sensor, with Gaussian noise augmentation improving the training robustness. Key features (rise time, decay time, peak width, decay slope) were selected via extremely randomized trees. Machine learning models (naïve Bayes, decision trees, logistic regression, KNN, and histogram-based gradient boosting) were trained with grid search optimization. The system achieved 88% accuracy for binary classification, 75–100% accuracy for multilabel classification, and near-perfect regression performance (R^2^ ≈ 1). The sensor detected VOCs at 50 ppb, maintaining stability under humidity and CO_2_, highlighting its potential for early, non-invasive diagnostics. Figure 3 summarizes the above studies.

Furthermore, Cao et al. [71] explored the use of volatile organic compounds (VOCs) in skin sebum as non-invasive biomarkers for Parkinson’s disease (PD). By employing an artificial intelligence olfactory (AIO) system that integrates gas chromatography (GC) with a surface acoustic wave (SAW) sensor, the study highlights a novel, cost-effective approach for diagnosing PD. This methodology underscores the potential of combining chemical analysis with artificial intelligence to transform clinical diagnostics while acknowledging the need for further research to refine disease progression stratification. Skin sebum samples were collected from 121 PD patients and 129 healthy controls to serve as the primary dataset. The computational pipeline included preprocessing the sebum samples to extract the VOCs, followed by data collection through the AIO system. Machine learning models, including gradient boosting decision tree (GBDT), random forest (RF), and extreme gradient boosting (XGB), were applied to classify PD patients and healthy controls. Validation of these models involved metrics such as the sensitivity, specificity, and area under the curve (AUC), ensuring robust assessment of the diagnostic performance. The results showed that the GBDT model outperformed the others, achieving a sensitivity of 83.33%, specificity of 84.00%, and an AUC of 0.893. Despite its success in identifying PD cases, the study faced challenges in predicting disease severity based on the Hoehn–Yahr scores.

Similarly, Choudhury et al. [75] investigated non-invasive biomarker identification, focusing on the potential of salivary biomarkers like S100A8 (calgranulin A) for diagnosing rheumatoid arthritis (RA). By integrating computational tools with proteomics, the study highlights a patient-friendly diagnostic approach, aiming to minimize invasive procedures while advancing autoimmune disease management. The computational pipeline included DEP identification using SWATH-MS for saliva samples from RA patients and healthy controls. Microarray datasets (GSE93272 for blood and GSE1919 for synovial tissue) were analyzed for differentially expressed genes (DEGs), with comparative analyses pinpointing S100A8 as a shared biomarker. Protein–protein interactions involving S100A8 were explored using STRING, while molecular docking with PyRx and the FT site server identified active sites and plant-based inhibitors with therapeutic potential.

Additionally, Wang et al. [48] investigated circulating plasma mRNAs as non-invasive biomarkers for prostate cancer (PCa) diagnosis. Highlighting key mRNAs such as PCA3, DLX1, DUOX1, and GSTP1, the study integrates bioinformatics and molecular techniques to identify plasma-based biomarkers, presenting a promising alternative to invasive diagnostic methods. These findings emphasize the potential of mRNA biomarkers in enhancing early detection and warrant further exploration in larger-scale studies. The initial biomarker discovery relied on microarray data from the Oncomine database, comparing gene expression in PCa tissues to adjacent normal tissues. The computational pipeline began with retrieving and analyzing gene expression data from the Oncomine database. Differentially expressed genes (DEGs) were identified using stringent *p*-value and fold-change criteria. Plasma mRNAs corresponding to these DEGs were extracted and quantified via qRT-PCR, followed by statistical analyses, including Mann–Whitney U tests and receiver operating characteristic (ROC) curve evaluations, to assess the biomarker performance. The analysis demonstrated that PCA3 and DLX1 were significantly overexpressed, while DUOX1 and GSTP1 were underexpressed in plasma samples from PCa patients compared to healthy controls. Diagnostic accuracy assessments indicated that DLX1 had the highest performance (AUC 0.821), followed by PCA3 (AUC 0.762), DUOX1 (AUC 0.7072), and GSTP1 (AUC 0.643). These outcomes highlight the strong potential of plasma-based mRNA biomarkers for early detection of PCa, offering a non-invasive diagnostic approach that can enhance clinical workflows.

Vasudevan et al. [24] introduced a novel framework for early cancer prediction, combining urinary biomarkers with machine learning models to identify pancreatic cancer in its early stages. Clinical data formed the backbone of the study, with the dataset incorporating features such as the age, sex, creatinine levels, and specific biomarkers, including LYVE1, REG1B, REG1A, and TFF1. The computational pipeline relied on gradient boosting algorithms, particularly XG-Boost and LightGBM, for predictive modeling. Preprocessing included data cleaning and feature selection to optimize model performance. The LightGBM model was noted for its computational efficiency and strong prediction accuracy. Cross-validation ensured robustness, while performance metrics such as the accuracy, recall, precision, and F1-score provided a comprehensive evaluation of the model efficacy. Among the models tested, XGBoost achieved the highest accuracy of 90.36%, while LightGBM demonstrated superior recall (88.32%) and precision (88.24%), showcasing its reliability in identifying pancreatic cancer cases. Biomarkers such as TFF1 and LYVE1 exhibited strong associations with cancer stages, underscoring their diagnostic relevance.

Furthermore, Charuet et al. [76] explored non-invasive biomarker identification for diagnosing significant liver fibrosis. By leveraging clinical, demographic, and laboratory data, the study emphasizes the potential of ensemble machine learning techniques, particularly the superlearner algorithm, to replace invasive procedures like liver biopsy. Data for the study were derived from three major cohorts: the NASH-CRN observational study, the FLINT trial, and the NHANES survey. The datasets included variables such as liver stiffness measurements obtained via transient elastography for the NHANES cohort, alongside clinical and demographic parameters. These datasets reflect a diverse population, providing a robust foundation for developing and validating the model. The computational approach employed the superlearner algorithm, which integrates predictions from 12 base models, including random forests, support vector machines, generalized linear models, and neural networks. Cross-validation ensured optimal weighting of the base models to maximize performance. The algorithm was trained and validated across the three datasets, with the AUC-ROC serving as the primary metric for assessing its predictive accuracy. The superlearner exhibited strong diagnostic capabilities, achieving AUCs of 0.79 in the FLINT cohort and 0.74 in the NHANES cohort for predicting stage 2 or higher liver fibrosis. It surpassed many existing fibrosis prediction tools, including FIB-4, APRI, and BARD, and performed comparably to the SAFE score in specific cases.

The identification of circulating miRNAs as non-invasive biomarkers for colorectal cancer (CRC) has also been examined [49]. By leveraging bioinformatics methodologies and publicly available datasets, the study highlights the stability of these miRNAs in serum, positioning them as promising candidates for cost-effective and non-invasive diagnostic tools. This comprehensive approach underscores the potential of circulating miRNAs to transform early detection strategies for CRC. The study utilized serum samples analyzed through microarray technology, with data sourced from the GEO database (GSE59856). The dataset included 50 CRC patients and 150 healthy controls, providing a robust foundation for miRNA expression profiling. Orthogonal partial least squares (OPLS) analysis identified DEMs, while support vector machine (SVM) models were constructed to evaluate the diagnostic accuracy. Two models were developed: one using all 569 DEMs and another employing the top three miRNAs with the highest weight coefficients. Additional analyses included protein–protein interaction (PPI) networks, gene regulatory networks (GRN), and pathway enrichment to contextualize the findings. The analysis revealed 569 DEMs, including 316 downregulated and 253 upregulated miRNAs in CRC patients. The SVM models achieved remarkable classification accuracy, with the primary model demonstrating an AUC of 1 and the simplified model achieving an AUC of 0.99. The PPI networks identified 110 hub genes associated with CRC, enriched in over 1000 biological processes and pathways. Furthermore, transcription factors such as STAT1 and CEBPD were identified as master regulators, providing deeper insights into CRC’s molecular mechanisms.

Mansouri et al. [77] explored non-invasive biomarkers for diagnosing endometriosis by examining immune factors, such as cytokines and immune cell markers, in peripheral blood. Employing machine learning to enhance the diagnostic precision, the study presents a compelling alternative to invasive laparoscopy, emphasizing the potential of immune profiling to transform endometriosis management. Serum samples from 321 patients undergoing diagnostic laparoscopy formed the basis of the study. The immune profiling included a range of cytokines (e.g., IL-10, IL-17, and IFNg), chemokines like CXCL1, T cell markers, and natural killer (NK) cell activity. By identifying changes in immune markers, the study underscores the practicality of using blood-based diagnostics to detect endometriosis, minimizing the need for invasive procedures. The computational methodology involved rigorous statistical analysis to identify differentially expressed immune markers, followed by machine learning modeling to classify patients. Logistic regression was employed, with the data divided into training and testing cohorts and validated through 3-fold cross-validation. Fine-tuning ensured optimal predictive accuracy, while independent test cohort validation demonstrated the model’s generalizability. The study revealed a tolerogenic immune profile in endometriosis patients, including increased IL-10 production in Tc cells, reduced IFNg production in NKT cells, decreased Treg cell levels, and diminished NK cytotoxic activity. Elevated CXCL1 levels, linked to IL-17 activity, further characterized this profile.

Additionally, Shibin et al. [53] focused on the identification of non-invasive biomarkers using surface-enhanced Raman scattering (SERS) technology. By combining serum biomarker detection with a novel Ag_2_O–Ag-porous silicon Bragg mirror (PSB) substrate and machine learning algorithms, the study demonstrates a promising approach for diagnosing autoimmune diseases such as Sjögren’s syndrome and diabetic nephropathy. This innovative framework underscores the potential of integrating advanced spectroscopy with computational modeling for rapid and cost-effective diagnostics. The study relied on serum samples obtained from blood, analyzed through SERS spectroscopy to detect disease-specific biochemical signatures. The computational pipeline incorporated preprocessing techniques such as baseline correction and smoothing using the airPLS and Savitzky–Golay algorithms. Principal component analysis (PCA) reduced the dimensionality of the spectral data, enabling efficient feature extraction. Classification was performed using a support vector machine (SVM), and the diagnostic performance was assessed through metrics like the sensitivity, selectivity, accuracy, and AUC. The findings highlight the effectiveness of the PCA-SVM framework combined with the composite SERS substrate. For Sjögren’s syndrome, the model achieved an accuracy of 90.7%, with a sensitivity of 93.4% and an AUC of 0.900. For diabetic nephropathy, the accuracy reached 89.3%, with a sensitivity of 95.6% and an AUC of 0.878.

Furthermore, Rehan et al. [50] investigated the non-invasive identification of biomarkers for diabetes mellitus, focusing on the urinary glucose and tryptophan levels. Utilizing fluorescence spectroscopy and hierarchical cluster analysis (HCA), the study explored the diagnostic potential of these biomarkers, offering a novel approach to diabetes detection without invasive procedures. This work highlights the integration of spectroscopic techniques and unsupervised machine learning to improve biomarker-based diagnostics. The study analyzed urine samples from 40 participants, split evenly between diabetic and non-diabetic controls.

Lastly, a study [73] investigated the volatile organic compounds (VOCs) in exhaled breath as non-invasive biomarkers for liver disease diagnosis, particularly cirrhosis. Using thermal desorption–gas chromatography–field asymmetric ion mobility spectrometry (TD-GC-FAIMS), breath samples from 50 participants were analyzed with machine learning models. Feature extraction identified the molecular signatures, with logistic regression computing a molecular feature score. Random under-sampling boosted trees (RUSBTs) and Gaussian naïve Bayes (GNB) were used for classification. The molecular feature score achieved 90% sensitivity and 57% specificity, while the RUSBT improved the specificity to 75%, with 88% sensitivity. Tandem models reached 89% accuracy for cirrhosis detection and 84% for decompensated disease classification, highlighting VOCs’ potential for liver disease diagnostics.

### 3.3. Signal-Data-Based Studies

To begin with the signal-data-based studies, Wang et al. [55] explored the potential of non-invasive biomarker identification through electrocardiographic features, aiming to detect prediabetes. The dataset comprised 12-lead ECG recordings, each lasting 5 s, collected from 2251 training cases and 663 independent testing cases. These recordings were processed into 2D image formats for analysis using deep learning techniques. To address the class imbalances and improve the model generalizability, data augmentation methods such as cropping and resizing were applied, effectively expanding the dataset. The IGRNet model delivered strong diagnostic performance, achieving an accuracy of 85.4%, sensitivity of 86.2%, and specificity of 86.5% on mixed test datasets. Additionally, the model excelled in BMI-specific datasets, particularly among individuals with a BMI below 25, where the validation accuracy increased significantly.

Additionally, Torshizi et al. [56] developed a non-invasive method for detecting hyperkalemia using ECG-derived features, eliminating the need for blood sampling. ECG data from 126 hyperkalemic patients and 152 controls were collected using a 12-lead Philips ECG device, with the analysis focused on lead 2. A computational pipeline extracted 16 critical ECG features, with principal component analysis (PCA) reducing the dimensionality. Machine learning models, including random forest (RF), decision tree (DT), SVM, and logistic regression (LR), were tested, with RF achieving the best performance (74% accuracy, 83% precision, 54% recall, AUC 0.69). While promising, further validation on larger populations is needed to enhance the diagnostic accuracy and generalizability.

Moreover, the potential of EEG signals as non-invasive biomarkers for diagnosing pediatric tic disorders (TD) was explored [78]. This approach emphasizes improving the diagnostic accuracy and objectivity, moving beyond traditional clinical methods through advanced computational analysis of brain activity. A one-dimensional convolutional neural network (1D-CNN) was implemented for feature extraction, coupled with max pooling to streamline the dimensionality. Residual neural networks (ResNets) were employed to extract deep features through residual blocks and bottleneck structures, enhancing the computational efficiency. The final classification was handled by a multi-layer perceptron (MLP) using softmax for probabilistic predictions. The performance evaluation involved four-fold cross-validation and metrics such as precision, recall, F1-score, and AUC. The ResNet model achieved an accuracy of 87.23%, with a precision of 88.47% and an AUC of 0.96, demonstrating strong diagnostic capability. Notably, the EEG signal regions along the Fp2-F4-C4 axis were identified as critical to the diagnostic predictions, emphasizing the importance of the frontal and central brain regions. While the study highlights the promise of EEG-based diagnostic tools for clinical use, challenges related to signal instability in extended recordings suggest further refinement is needed for robustness.

In a similar vein, Ma et al. [69] developed a non-invasive method for diagnosing CHD-associated pulmonary arterial hypertension (CHD-PAH) using heart sound signals (PCG). A computational pipeline processed the heart sounds, isolating the second heart sound (S2), a key diagnostic marker. Feature extraction included time-domain (cycle intensity, intervals), frequency-domain (spectral entropy, dominant frequency), and deep learning-based PNCC features via a CNN. The features were fused into a single vector and classified using XGBoost, with majority voting enhancing the accuracy. The model achieved 88.61% accuracy for three-class classification (normal, CHD, CHD-PAH), demonstrating the effectiveness of integrating multiple feature extraction techniques for non-invasive cardiac diagnosis.

Similarly, Liu et al. [57] developed a non-invasive method for diagnosing fetal arrhythmias using non-invasive fetal electrocardiography (NI-FECG) signals. Data from the NIFEADB database were processed through a computational pipeline, including noise removal, segmentation, and multi-domain feature extraction (wavelet entropy, sample entropy, and Hilbert–Huang transform). Neighborhood component analysis (NCA) reduced the features from 120 to 40, optimizing the classification. A hierarchical extreme learning machine (H-ELM) framework, integrating ELM-SAE for feature extraction and an ELM classifier, outperformed SVM, random forest, and DBN, achieving 96.33% accuracy, 99.11% sensitivity, and 93.91% specificity. While the model demonstrated strong performance, further validation on diverse datasets is needed.

In a similar vein, Duc et al. [54] conducted a non-invasive approach for diagnosing type 2 diabetes mellitus (T2DM) by combining Raman spectroscopy data with advanced machine learning models. Data for the study were collected in vivo from 20 participants, including 11 T2DM patients and nine healthy controls. Raman spectroscopic scans targeted skin sites such as the cubital vein, earlobe, inner arm, and thumbnail. Preprocessed spectral data, with fluorescence background subtraction and normalization, formed the basis of the computational analysis. Dimensionality reduction through principal component analysis (PCA) retained 99% of the data’s variance, ensuring robust feature representation. To classify the data, the study utilized support vector machines (SVMs) and artificial neural networks (ANNs). Hyperparameter tuning optimized both models, with the ANN incorporating two hidden layers and leveraging activation functions like ReLU, tanh, and sigmoid. Five-fold cross-validation ensured robust evaluation of the performance metrics, including the accuracy, sensitivity, specificity, and ROC-AUC scores. Among the models tested, the ANN outperformed the others, with an accuracy of 93.8% and a ROC-AUC of 0.96, highlighting its superior diagnostic capabilities. Measurements taken from the cubital vein emerged as the most reliable, offering optimal diagnostic precision.

**Figure 4:**
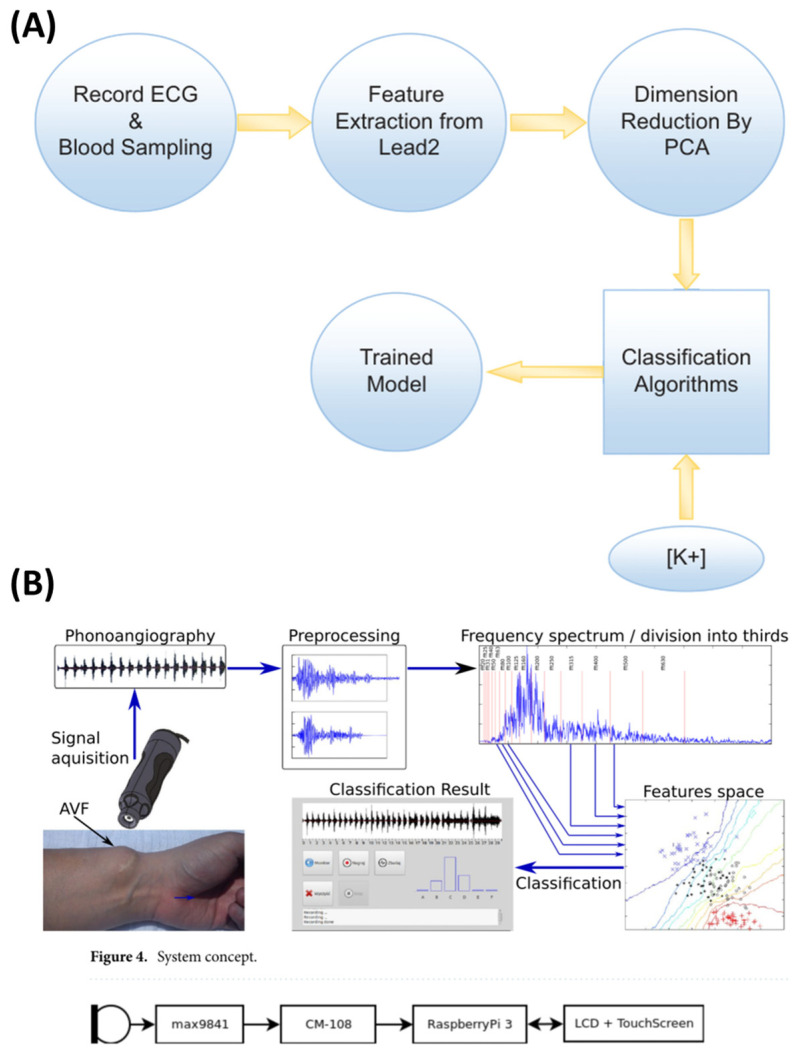
Collage of 2 indicative signal-based non-invasive methodologies. (**A**) A general chart outlining the study steps of Torshizi et al. [56] and (**B**) an overview of the hardware layer structure by Grochowina et al. [59].

Similarly, Grochowina et al. [59] introduced a prototype device and machine learning framework designed for non-invasive monitoring of arteriovenous fistula (AVF) conditions in hemodialysis patients. By utilizing acoustic signals as biomarkers, this approach targets vascular access stenosis with enhanced diagnostic precision. The data comprised acoustic recordings from the AVFs of 38 chronic hemodialysis patients. The acoustic signals were analyzed using fast Fourier transform (FFT), extracting 23 features from the time-domain waveforms and frequency spectrums. Feature selection methods such as PCA, forward search, and feature-class correlation refined the dataset for optimal predictive accuracy. Supervised machine learning algorithms, including K-nearest neighbor (KNN), support vector machine (SVM), and random forest, were implemented, with KNN yielding the best results. Leave-one-out cross-validation ensured the robustness and generalizability of the system. Achieving an 81% classification accuracy across six stages of stenosis progression, the KNN-based system demonstrated clear potential as a non-invasive and reliable diagnostic tool for AVF monitoring. Figure 4 shows signal-based non-invasive methodologies.

In addition, the potential of non-invasive biomarkers derived from 12-lead electrocardiograms (ECGs) to predict HbA1c levels and diagnose diabetes mellitus was explored [58]. The research utilized 29,353 unique ECG recordings linked to HbA1c measurements from 5570 adult patients. The ECG signals were processed using a convolutional neural network (CNN) to investigate their utility in distinguishing between diabetes, prediabetes, and normal glycemic states. Noise reduction techniques, including median filtering and signal averaging, were applied to refine the ECG data. This dataset, split into training and validation cohorts in an 80:20 ratio, was used to develop and evaluate the AI model. Analysis revealed that the ECG features alone provided limited predictive accuracy for the HbA1c levels. While the AI model classified the diabetes-related categories with moderate C-statistics of 0.63 (no diabetes), 0.51 (prediabetes), and 0.59 (diabetes), the overall accuracy and F1 scores hovered around 0.43. These findings suggest that ECG-derived biomarkers, in isolation, lack sufficient discriminatory power for reliable diabetes screening. Incorporating additional predictors and expanding dataset diversity are proposed as necessary steps for improving future AI-driven diagnostic frameworks.

Last but not least, Hu et al. [70] introduced a novel diagnostic framework for coronary heart disease (CHD). The study developed a light-activated virtual sensor array (LAVSA) utilizing black phosphorus (BP) and Ti3C2Tx MXene composites to analyze the volatile organic compounds (VOCs) in exhaled breath, enabling non-invasive biomarker detection. Data for this research came from gas signals extracted from breath samples of 45 individuals, encompassing both healthy participants and CHD patients. The methodology integrated material science with computational approaches. The LAVSA sensor captured breath VOCs with high sensitivity, detecting concentrations as low as 50 ppb. Pattern recognition techniques such as principal component analysis (PCA) and t-distributed stochastic neighborhood embedding (t-SNE) facilitated the initial data classification. Machine learning algorithms, including logistic regression, K-nearest neighbor (KNN), and support vector machines (SVM), were then applied to train and validate the predictive models for CHD diagnosis. The findings highlighted the system’s capacity to classify CHD patients and differentiate stenosis levels with 69.2% accuracy.

### 3.4. Clinica-Data-Based Studies

Building on the use of non-invasive clinical data for biomarker identification, Rishika et al. [61] explored non-invasive methods for detecting congenital heart disease (CHD) using patient-level data. Data from 36,300 patient records formed the foundation of this research. The features included the maternal age, family history of birth defects, nutritional deficiencies, and exposure to risk factors during pregnancy. The structured tabular dataset provided a robust base for predictive analysis without relying on imaging, molecular techniques, or wearable devices. The computational pipeline encompassed preprocessing steps to handle missing data, which were imputed using the mode values for each attribute. A suite of machine learning algorithms, including logistic regression, support vector machine, random forest, and XGBoost, were tested alongside a deep learning model. The proposed neural network architecture featured two dense layers with ReLU activation for the hidden layers and sigmoid activation for binary classification. Performance metrics such as the accuracy, precision, recall, F1 score, and execution time were used to evaluate the models. The predictive models showcased impressive performance. Machine learning approaches, including ANN and XGBoost, consistently achieved an accuracy of 99.79% for CHD detection. The execution times varied, with the ANN requiring the longest time of 1129.8 s, while Gaussian naïve Bayes completed the task in just 0.024 s.

Moreover, a non-invasive framework for dementia diagnosis using qualitative data derived from conversational surveys was proposed [62]. The dataset used was sourced from the Behavioral Risk Factor Surveillance System (BRFSS), a comprehensive health survey conducted by the CDC. This dataset includes records of demographics, functional impairments, and subjective cognitive assessments, providing a qualitative foundation for analysis. The computational pipeline employed a chatbot interface powered by NLP to facilitate cognitive assessments through surveys. The responses were transformed into quantitative variables for predictive analysis, and various machine learning models, including random forest, logistic regression, decision trees, and k-means clustering, were implemented. The model evaluation relied on metrics like the area under the precision–recall curve (AUC-PR) and Matthew correlation coefficient (MCC) to ensure robustness. Among the models tested, the random forest algorithm delivered the most promising results, achieving an AUC-PR score of 0.83 and an MCC of 0.53. Subjective cognitive decline emerged as the most significant predictor, followed by functional difficulties and demographic factors. These findings underscore the potential of leveraging conversational data for accessible and effective dementia diagnosis. Table 3 summarizes the distinct computational methods, algorithms, and tools that are recurrently applied, shedding light on the analytical frameworks supporting non-invasive biomarker exploration.

In a similar vein, Wang et al. explored non-invasive cognitive impairment diagnostics through the use of language-based digital markers [60]. The study leverages speech and cognitive tasks to extract interpretable features that aid in the early detection of cognitive decline, offering a scalable and accessible diagnostic tool. The methodology involved multiple steps, including extracting acoustic features like the silence duration and pause-to-speech ratios. The linguistic analyses incorporated part-of-speech tagging and graph metrics to explore the semantic relationships. Machine learning models such as logistic regression, random forests, and support vector machines were deployed within the SHAP framework to ensure feature interpretability. The models were further tested for cross-lingual applicability with data in both Chinese and English, as validated on independent cohorts for generalizability. Significant findings emerged, with the models achieving accuracy rates of 83% for differentiating normal cognition, amnestic mild cognitive impairment (aMCI), and Alzheimer’s disease (AD). Cross-lingual robustness was demonstrated with an accuracy of 76%, while independent cohort validation showed a prediction accuracy of 75%, with sensitivities and specificities above 68%.

Furthermore, the examination of the clinical outcomes related to hypertensive disorders of pregnancy (HDP) in infertile patients was performed, categorizing cases based on the mode of conception: unassisted conception (UA), non-IVF treatment (NIFT), and assisted reproductive technology (ART) [79]. Insights from this work emphasized the importance of patient demographics and clinical history in understanding HDP risks. Clinical data from 625 singleton pregnancies were analyzed, with patients grouped into the UA, NIFT, and ART categories. Parameters such as gestational hypertension, preeclampsia, age, BMI, chronic hypertension, diabetes, and nulliparity were evaluated. Structured clinical records formed the basis of the analysis, without integrating molecular data or wearable devices. No specific biological tissue was investigated. Instead, the focus remained on clinical outcomes and gestational factors. Statistical approaches, including chi-squared tests and ANOVA, compared the HDP diagnoses and gestational ages across groups, while additional risk factors like the BMI and age were analyzed with R software using a significance threshold of 0.05. No significant differences in the HDP incidence were observed across the conception methods, with a *p*-value of 0.66. The gestational hypertension and preeclampsia rates were similar in the UA, NIFT, and ART groups. The ART patients were slightly older, averaging 38.7 years, and the BMI differences, though statistically significant, were minor.

### 3.5. Combined Modality Studies

There are studies that combine data from multiple modalities to develop innovative diagnostic approaches for various diseases. In this vein, Shahrbabak et al. [68] presented a proof-of-concept study using synthetic data and deep learning techniques to establish the efficacy of non-invasive peripheral artery disease (PAD) diagnosis. This work emphasizes the potential of computational models integrated with non-invasive biomarkers like pulse volume recording (PVR) waveforms to offer accessible and affordable diagnostic solutions. The investigation centered on arterial pulse waveforms derived from the brachial and tibial arteries. These waveforms reflected the blood circulation dynamics rather than biological tissues like blood or saliva, offering a novel perspective on non-invasive diagnostics. The in silico methodology encompassed synthetic data generation using a multi-branch transmission line model to simulate realistic BP and PVR waveforms across varying PAD severity levels. The CNN, based on the AlexNet architecture with continuous property-adversarial regularization (CPAR), was trained to classify PVR waveforms. Comparative performance analysis between PVR waveforms, BP waveforms, and the conventional ankle-brachial index (ABI) highlighted the CNN’s diagnostic capabilities. Key metrics such as the sensitivity, specificity, accuracy, and the area under the ROC curve (AUC) were used for evaluation. The results demonstrated high diagnostic accuracy, with the CNN achieving an AUC of ≥0.89 using the PVR data, comparable to the BP data (AUC ≥ 0.96) and significantly outperforming the ABI (AUC ≤ 0.59). The positive and negative predictive values for PAD detection were robust, measuring ≥ 0.78 and ≥ 0.85, respectively. Although the CNN effectively detected PAD, its performance in estimating the PAD severity was moderate, with a correlation coefficient of 0.77.

Similarly, Beltran et al. [52] conducted an experimental study integrating machine learning approaches with biomarker analysis to predict the progression of Alzheimer’s disease (AD) from mild cognitive impairment (MCI). This work underscores the potential of plasma-based biomarkers as a cost-effective and non-invasive diagnostic alternative, particularly in the early stages of the disease. By incorporating advanced computational methods, the study enhanced the predictive accuracy, contributing to scalable diagnostic solutions. The research focused on identifying plasma biomarkers that could predict the transition from MCI to AD, leveraging machine learning models to develop accessible diagnostic methods. Drawing from the Alzheimer’s Disease Neuroimaging Initiative (ADNI) database, the study utilized datasets that included 145 plasma analytes, composite cognitive scores for memory and executive function, and demographic variables such as the age, BMI, and ApoE4 gene status. High-dimensional data were reduced using principal component analysis (PCA) for MRI data and prior knowledge-based selection of significant plasma biomarkers. Multiple machine learning models, including classification and regression trees (CARTs), random forests (RFs), gradient boosting (GB), and support vector machines (SVMs), were trained and evaluated using performance metrics like the area under the ROC curve (AUC), sensitivity, and precision–recall curve. Stringent validation methods, such as leave-two-out cross-validation and testing on separate datasets, ensured robust model evaluation. Integrating cognitive scores, demographic data, and plasma biomarkers yielded an AUC of approximately 0.75, with the addition of the MRI atrophy variables improving this to 0.77. The significant plasma biomarkers included apolipoproteins, leptin, insulin, and CRP, all of which were identified as critical predictors of disease progression.

Additionally, an experimental study focused on non-invasive biomarker identification for staging liver fibrosis was executed [63]. Their work investigated serum markers and patient characteristics, such as the liver enzyme levels and RNA markers, to replace the need for invasive liver biopsies. By leveraging machine learning techniques, the study highlights a promising approach for diagnosing liver fibrosis in a non-invasive and cost-effective manner. The research utilized data from a cohort of 1385 patients undergoing hepatitis C virus (HCV) treatment over 15 months. This dataset included serum markers like alanine transaminase (ALT), aspartate transaminase (AST), and RNA levels at various treatment weeks, as well as patient demographics such as age, BMI, and clinical symptoms like fatigue and jaundice. The computational pipeline involved several steps, starting with data preprocessing techniques like one-hot encoding for categorical variables and Z-score standardization for continuous variables. Feature selection was performed using a random forest classifier to identify the most relevant variables contributing to model accuracy. Classification models, including logistic regression, random forest, and multi-layer perceptron (MLP), were applied to predict fibrosis stages. Additionally, a decision tree algorithm was used to generate interpretable diagnostic rules. The models were rigorously validated through five-fold stratified cross-validation to ensure their reliability. The MLP model demonstrated exceptional predictive accuracy, achieving 97.83% with the full feature set and 97.40% with a reduced feature set. The random forest and logistic regression models also performed well, with accuracies exceeding 97%. The RNA levels measured after four weeks of treatment emerged as the most significant predictor, while the ALT and AST levels were crucial for distinguishing between stages like portal fibrosis and cirrhosis. The study also generated a concise set of 28 interpretable rules for staging fibrosis, simplifying the diagnostic process compared to earlier models with over 98,000 rules.

Moreover, Jain et al. [72] presented an experimental study that combined IoT technologies, artificial intelligence, and deep learning to develop a secure, non-invasive diagnostic framework for COVID-19. This innovative approach integrates non-invasive tools, such as thermal scanners and pulse oximeters, with advanced machine learning techniques to detect early biomarkers indicative of COVID-19 infection. The research utilized data from thermal imaging, oxygen saturation levels measured through pulse oximeters, and chest X-ray images. These datasets were processed using IoT devices and analyzed through deep learning models to enhance the diagnostic accuracy. The computational pipeline for the study was structured into three distinct phases. Initially, an AI-enabled chatbot was employed to collect symptom information from users. This was followed by non-invasive physiological measurements, including body temperature and oxygen saturation, using IoT devices such as thermal scanners and pulse oximeters. In the final phase, chest X-ray data were analyzed using two deep learning models: COVID-ConvNet and COVID-CapsNet. The ConvNet model utilized convolutional layers for feature extraction, while the CapsNet model leveraged capsule networks to preserve the spatial hierarchies, enhancing the classification accuracy. Both models were trained and validated on relevant datasets, with the performance evaluated through the precision, recall, and accuracy metrics. The findings revealed that COVID-CapsNet outperformed COVID-ConvNet, achieving an impressive accuracy of 97%, compared to 86% for the ConvNet model. The framework demonstrated significant potential in identifying COVID-19 in its early stages by combining IoT-enabled diagnostic tools with AI-driven analysis.

In addition, Hu et al. [41] introduced an experimental framework that integrates circulating tumor cells (CTCs) from liquid biopsies with multimodal magnetic resonance imaging (MRI) radiomics to enhance glioma diagnosis and molecular classification. This innovative approach combines non-invasive diagnostic tools to address both tumor grading and molecular subtyping, offering a precision medicine alternative for patients unsuitable for invasive procedures. The research utilized MRI data from preoperative scans sourced from the BraTS 2019 database and proprietary datasets from two medical centers. In addition, liquid biopsy data were derived from peripheral blood samples analyzed for CTCs. These datasets were fed into a multi-task deep learning radiomic model to achieve comprehensive diagnostic outcomes. The MRI preprocessing included segmentation through CSPDarkNet-tiny-based models, skull stripping, and bias field correction, ensuring high-quality radiomic feature extraction. The computational model combined these radiomics with CTC-derived numerical data for glioma grading and molecular classification, such as IDH mutations and ATRX deletions. The peripheral blood samples underwent CTC enrichment and molecular characterization through MALBAC single-cell amplification and Illumina sequencing. The framework achieved notable success in preoperative glioma grading and molecular subtyping by effectively integrating imaging and liquid biopsy features. While the specific performance metrics were not fully detailed, the study demonstrates the potential of combining radiomics and molecular diagnostics to improve the precision and reliability in glioma diagnosis.

Cook et al. [42] presented an innovative approach to non-invasive biomarker identification, introducing the TumorIO biomarker. This computational tool integrates biophysical features derived from dynamic contrast-enhanced magnetic resonance imaging (DCE-MRI) with transcriptomic data to predict responsiveness to immune checkpoint inhibitors (ICIs). By combining imaging and transcriptomics, the research addresses the need for precise, personalized approaches to immunotherapy. The study utilized DCE-MRI scans and RNA-seq data from breast cancer patients enrolled in the I-SPY1 and I-SPY2 clinical trials. The imaging data underwent biophysical simulations to extract the spatial and temporal tumor features, while the transcriptomic analyses focused on genes associated with metabolic and angiogenic activity. The computational framework relied on a biophysics simulation platform, integrating imaging data with RNA-seq results to characterize the tumor microenvironment comprehensively. The tumor tissue and the surrounding microenvironment were analyzed through imaging and biophysical simulations, capturing dynamic changes in the blood flow, nutrient delivery, and immune activity. The computational pipeline included the analysis of transcriptomic data to identify key metabolic and angiogenic genes, biophysical modeling of DCE-MRI data using the Simul-omics 4D Engine, and the development of the TumorIO Score. This score, based on a linear regression model, was validated using a small patient cohort and virtual clinical trials to ensure predictive accuracy. The TumorIO biomarker demonstrated exceptional performance, predicting a pathologic complete response (pCR) in 88.2% of an independent cohort. Specifically, 10 out of 12 predictions were accurate in triple-negative breast cancer (TNBC), and all 5 predictions were correct in hormone-receptor-positive (HR+)/HER2- tumors. Virtual clinical trials using TumorIO closely mirrored the real-world outcomes, with predicted pCR rates of 67.1% for TNBC and 17.9% for HR+/HER2- tumors.

Last but not least, [51] Hou et al. explored a groundbreaking approach to diagnosing glomerular diseases using hyperspectral imaging and artificial intelligence. By focusing on urine as a non-invasive biofluid, the study highlights the potential of leveraging spectral data to classify specific glomerular diseases accurately. Urine samples were collected from 65 patients diagnosed with idiopathic membranous nephropathy, minimal change nephropathy, IgA nephropathy, and diabetic nephropathy. Hyperspectral imaging was employed to capture spectral information ranging from 400 to 1000 nm, transforming the urine samples into digitized 2D spatial–spectral images. These data enabled detailed spectral analysis to differentiate disease-specific patterns effectively. A 34-layer residual network (ResNet) was constructed to analyze the spectral data. The model was trained and validated using 84,000 spectral images, with 80% allocated to training and 20% reserved for testing. Statistical analyses, including the Kruskal–Wallis H test, were performed to validate the spectral differences across disease types. Performance metrics such as the sensitivity, specificity, and ROC curves were used to evaluate the model’s diagnostic accuracy. The model achieved exceptional diagnostic accuracy of 96%, with sensitivity values ranging from 94% to 97% and specificity exceeding 98%. The AUC for the test set was similarly high at 96%, demonstrating the robustness of the approach. The significant spectral differences among the disease groups further validated the potential of hyperspectral imaging as a diagnostic tool.

## 4. Discussion

The analysis of the investigated studies reveals a predominant focus on imaging and molecular data as the primary sources for biomarker identification. These data modalities are frequently leveraged due to their capacity to provide comprehensive insights into disease mechanisms and states. A noteworthy observation is the significant emphasis on utilizing machine learning (ML) and artificial intelligence (AI) tools for biomarker discovery, interpretation, and potential diagnostic applications. Both classical ML approaches and more advanced deep learning methods have been extensively applied, highlighting their pivotal role in advancing the field [80].

**Figure 5:**
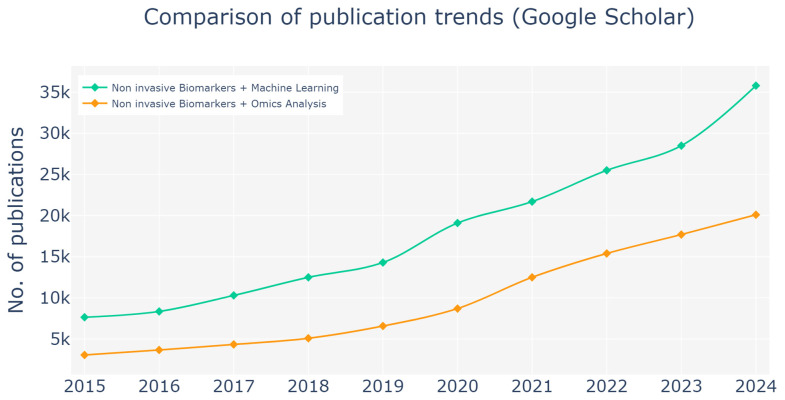
Publication trends from 2015 to 2024 (search on Google Scholar), highlighting the increasing focus on non-invasive biomarker methodologies. The analysis is centered on publications involving the terms “Noninvasive Biomarkers + Machine Learning” and “Non-invasive Biomarkers + Omics Analysis”. The observed rise in publications underscores the growing interest in these approaches within the field of biomarker research. A sharper increase is evident in studies referencing machine learning, reflecting its expanding role in advancing non-invasive biomarker applications. These trends emphasize the significance of both omics analyses and machine learning models and their potential in the development and enhancement of non-invasive diagnostic and prognostic tools.

This trend aligns with the publication patterns depicted in Figure 5, which illustrates the growing research interest in non-invasive biomarker methodologies over the past decade. An analysis of Google Scholar publications from 2015 to 2024 demonstrates the increasing prevalence of studies incorporating terms such as “Non-invasive Biomarkers + Machine Learning” and “Non-invasive Biomarkers + Omics Analysis”. The data reveal a sharper rise in publications referencing “machine learning”, underscoring its expanding influence on enhancing the precision and applicability of non-invasive biomarker techniques. These trends emphasize the dual importance of omics analyses and machine learning models in shaping the future of non-invasive diagnostics and prognostics, reaffirming their transformative potential in the development of advanced diagnostic tools.

Moreover, non-invasive biomarkers have markedly transformed clinical practice by enhancing the precision and efficiency of disease diagnosis and management. For instance, clinical prediction rules such as FIB-4, in combination with serum-based and elastography-based assessments, have become essential tools in the early risk stratification of liver diseases, enabling clinicians to identify patients at higher risk of adverse outcomes more reliably. Similarly, advanced MRI-based biomarkers are now instrumental in monitoring treatment responses in early-phase non-alcoholic steatohepatitis trials, paving the way for more individualized therapeutic strategies. In parallel, the integration of non-invasive approaches in other clinical areas, such as lung cancer care, has contributed to more effective symptom management and improved quality of life. Overall, these innovations reduce the reliance on invasive diagnostic procedures, streamline clinical decision-making, and support a move toward precision medicine, thereby optimizing patient outcomes and resource allocation within healthcare settings [81,82].

Despite the promising potential of non-invasive biomarkers, several challenges must be addressed to facilitate their integration into routine clinical practice. One significant obstacle is the limited sensitivity and specificity of some non-invasive biomarkers compared to traditional invasive methods. These limitations can result in false positives or negatives, hindering their reliability for early diagnosis and accurate disease monitoring. Furthermore, the variability of biomarker levels across populations, influenced by factors such as age, gender, ethnicity, and lifestyle, adds complexity to establishing standardized thresholds and interpretations. This lack of uniformity can affect the reproducibility of results and complicate the development of universal diagnostic criteria.

In addition, the reliance on limited datasets presents a significant challenge in developing effective non-invasive diagnostic models for specific diseases. The use of small sample sizes and lack of diverse data sources may lead to models that do not adequately capture the full spectrum of patient variability, ultimately compromising both accuracy and reliability. This shortfall in data representation risks producing diagnostic tools that may function well under controlled conditions yet falter in real-world clinical settings, where patient demographics and disease presentations are far more heterogeneous.

Another challenge comes from artifacts introduced by the use of machine learning (ML) and big data techniques, particularly when integrating datasets collected under different conditions. As highlighted in the study by Javaid et al., the adoption of health informatics and AI-driven decision-making can be influenced by variations in data collection environments, infrastructure differences, and the heterogeneity of imaging technologies used across institutions. Similarly, Dash et al. emphasized that the effectiveness of big data analytics in healthcare depends on systematic data integration and proper handling of discrepancies arising from diverse data sources. For example, if healthy volunteer data are predominantly sourced from routine screenings at local clinics using standard equipment, while clinical data for more severe cases are obtained from hospitals with advanced imaging facilities, ML models may learn to rely on image quality differences or vendor-specific artifacts rather than genuine disease-related patterns. This issue underscores the necessity of incorporating metadata and demographic adjustments to mitigate potential biases and improve model generalizability [83,84].

The required data size for different studies varies significantly depending on the complexity of the research question, the analytical methods employed, and the nature of the data itself. Big data analytics in healthcare often involves integrating electronic health records, medical imaging, genomic data, and real-time sensor outputs from wearable devices. While some studies, such as population-wide epidemiological research, demand extensive datasets to capture diverse demographic and clinical variables, others, like targeted biomarker discovery, can achieve meaningful results with smaller but high-quality datasets. The scalability of the data infrastructure also plays a crucial role in determining the feasibility of large-scale machine learning models. A study on disease prediction using deep learning may require millions of samples to ensure robust generalization, whereas a focused investigation into the effects of a specific drug may rely on a much smaller, curated dataset. Understanding the relationship between dataset size, study objectives, and computational constraints is essential for optimizing the balance between data volume and analytical rigor.

Another key challenge lies in the technological and computational aspects of non-invasive biomarker research. The analysis of multi-modal data, including omics datasets and imaging results, often requires advanced ML and AI tools, which can be resource-intensive and demand significant expertise. Additionally, there are concerns regarding data quality, as non-invasive samples like saliva or breath can be affected by external contaminants or inconsistent collection protocols. Ensuring robust validation of biomarkers across diverse clinical settings and populations is essential to overcome these hurdles. Addressing these challenges through rigorous research, standardization, and technological advancements may be critical to fully exploiting the potential of non-invasive biomarkers in precision medicine.

## 5. Conclusions

Non-invasive biomarkers are increasingly important for managing chronic conditions and conducting large-scale health screenings, offering the advantage of more frequent and accessible testing while reducing the risks associated with traditional procedures. This shift underscores the demand for diagnostic strategies that prioritize ease of use, patient well-being, and cost-efficiency, without sacrificing diagnostic precision. The integration of cutting-edge computational technologies, including high-throughput big data analytics through AI and ML techniques, is revolutionizing clinical practices. These enhancements help in recognizing novel patterns and predictive biomarkers in vast, multi-dimensional datasets. As a result, non-interventional approaches are transforming the field of disease detection and monitoring, enabling earlier, more accurate diagnoses and personalized treatment plans for patients.

## Figures and Tables

**Table 1:** Prevalent data modalities for the identification of various types of non-invasive biomarkers. The present recording was conducted between 2019 and 2024.

Data Type	Modality	Description	Reference(s)
Imaging	Ultrasound	Deep learning and computational models, including segmentation-based bi-attention DoubleUNet, compression ultrasound interpretation networks, 0D cardiovascular simulations, and AlexNet-based classifiers, were employed to enhance the non-invasive diagnosis of vascular, hepatic, and pancreatic conditions.	[29,30,31,32]
	Dermoscopy	Multi-layer machine learning (decision trees, random forests, SVM, KNN) and deep learning ensembles (MobileNet, Xception, ResNet50, DenseNet121) for melanoma diagnosis using dermoscopic images.	[23,33]
	RGB	Support vector machine (SVM), K-nearest neighbor (KNN), decision tree, and discriminant analysis models applied to RGB and thermal imaging data for non-invasive pneumonia diagnosis and therapeutic monitoring.	[34]
	CT scan	Deep learning and machine learning models, including ResNet V2 with a multi-task autoencoder for CNS tumor diagnosis and radiomics-based random forest and gradient boosting machines for colorectal cancer prognosis, were applied to analyze CT scan images non-invasively.	[35,36]
	MRI	Various machine learning models were applied to MRI images, including ResNet V2 and a multi-task autoencoder for CNS tumor diagnosis, a multi-layer perceptron for Menière’s disease classification, deep learning radiomics with liquid biopsy for glioma diagnosis, CNNs for non-invasive disease detection, integrative biophysical modeling for breast cancer immunotherapy profiling, a non-local ResNet and MMoE for NAFLD assessment, SVM and logistic regression for uterine leiomyoma subtyping, and radiomics-based LDA and SVM for meningioma grading.	[31,37,38,39,40,41,42]
Molecular	Blood	Multi-task deep learning, deep and shallow neural networks, ResNet-18, and ensemble machine learning models were used for glioma diagnosis, colorectal cancer detection, bladder cancer classification, and coronary artery disease risk stratification using blood-based biomarkers.	[41,43,44,45]
	ctDNA/RNA/microRNA	Latent Dirichlet allocation (LDA) was used for microRNA biomarker discovery in prostate cancer, while computational frameworks integrating circulating microRNAs, cell-free DNAs, and proteins employed machine learning models for enhanced disease detection and monitoring.	[6,7,26]
	Microarrays/RNA-seq	Machine learning models, including SVM for colorectal cancer biomarker identification, CNN for NAFLD classification, bioinformatics-driven PPI network analysis for pancreatic cancer biomarkers, and differential expression analysis with machine learning for plasma mRNA-based prostate cancer detection, were applied to microarray and RNA-seq data.	[30,46,47,48,49]
	Urine	Deep and shallow neural networks for colorectal cancer diagnosis, ResNet-18 for bladder cancer classification using urine droplet patterns, hierarchical cluster analysis for glycosuria and diabetes biomarker identification, and a 34-layer residual network for non-invasive glomerular disease diagnosis using hyperspectral urine analysis were applied to urine-based data.	[43,44,50,51]
	Plasma	Random forest, gradient boosting, CART, and SVM were used for Alzheimer’s disease prediction based on plasma biomarkers, while bioinformatics-driven differential gene expression analysis and statistical machine learning identified novel plasma mRNA biomarkers for prostate cancer diagnosis.	[48,52]
Signal	Spectroscopy	PCA-SVM was used for serum feature extraction and disease classification, while a customized ANN and PCA-SVM were applied for non-invasive type 2 diabetes mellitus diagnosis using spectroscopy-based data.	[53,54]
	ECG	Deep learning and machine learning models, including IGRNet (CNN) for prediabetes diagnosis, random forest with PCA for hyperkalemia classification, hierarchical extreme learning machine (H-ELM) for fetal arrhythmia detection, and a CNN for HbA1c-based diabetes prediction, were applied to ECG-based data.	[55,56,57,58]
	Acoustic	KNN, SVM, and random forest were used for arteriovenous fistula classification with phono-angiography signals, while logistic regression, SVM, and random forest were applied for cognitive impairment diagnosis using cross-lingual speech features.	[59,60]
Clinical	Patient Record	Random forest, gradient boosting, CART, and SVM were used for Alzheimer’s disease prediction based on cognitive scores and genetic risk factors, ANN, random forest, XGBoost, AdaBoost, decision tree, naïve Bayes, logistic regression, and SGD for congenital heart disease diagnosis using electronic health records, a random forest model with NLP for dementia diagnosis using qualitative cognitive assessments, and multi-layer perceptron, random forest, and logistic regression for liver fibrosis staging using clinical parameters.	[24,52,61,62,63]

**Table 2:** Disease categories and conditions identified in the current work, reflecting both common and emerging trends.

Disease Class	Condition	Reference(s)
Cancer	Colorectal	[36,43,49]
	Skin	[23,33]
	NSCLC	[65]
	Pancreatic	[24,47]
	Bladder	[44]
	Prostate	[48]
	Glioma	[41]
	Breast	[42]
	Uterine Fibroid	[37]
Cardiovascular	Deep Vein Thrombosis	[29]
	Peripheral Artery Disease	[68]
	Coronary Heart Disease	[61,69,70]
	Arrhythmia	[57]
Neurodegenerative	Alzheimer’s Disease	[52,60]
	Parkinson’s Disease	[71]
	Dementia	[52,62]
Respiratory	Pneumonia	[34]
	Idiopathic Pulmonary Fibrosis	[64]
	COVID-19	[34,72]
Metabolic	Diabetic Nephropathy	[51,53]
	Diabetes Mellitus	[32,50,54,58]
Autoimmune	Sjogren’s Syndrome	[53]
Liver	Portal Fibrosis	[63]
	Septa	[63]
	Cirrhosis	[31,63,73]

**Table 3:** Summary of the computational methodologies and algorithms for non-invasive biomarker monitoring presented in the current study.

Computational Methodology	Algorithm	Reference(s)
Supervised ML	SVM	[22,33,34,49,53,54,56,59,60,61,66,73]
	Random Forest	[22,33,36,45,52,56,59,62,63,65,71]
	Decision Tree	[34,56,62,63,71,74]
	Gradient Boosting	[24,33,36,45,52,71]
	KNN	[24,34,61,73]
	Logistic Regression	[56,60,61,62,63,77]
	XGBoost	[24,61,69]
	LightGBM	[24]
	Gaussian Naive Bayes	[73]
	LASSO	[40]
	Linear Discriminant Analysis	[34,40]
	Quadratic Discriminant Analysis	[34]
	CART	[70]
	Ensemble	[22,33,76]
Unsupervised ML	K-Means Clustering	[62]
	Hierarchical Cluster Analysis (HCA)	[50]
	Gaussian Mixture Models	[35]
Deep Learning	ANN	[41,54,61]
	Convolutional Neural Networks	[29,39,46,68]
	Fully Convolutional Networks	[39]
	ResNet	[33,35,44,46,51,64,78]
	AlexNet	[32,55]
	CapsNet	[72]
	U-Net	[29,46]
	ResNeXt	[46]
	Masked Autoencoder	[65]
	Principal Component Analysis	[22,34,38,52,53,54,56,59,65,70]
	t-SNE	[70]
	Neighborhood Component Analysis	[57]
Dimensionality Reduction	Wavelet Entropy	[57]
	Hilbert–Huang Transform	[57]
	Fast Fourier Transform	[59]
Feature Selection and Extraction	SHAP	[35,40,45,60]
	Saliency Maps	[35]
	Bioinformatics pipeline	[48,49]
Other	Cytoscape	[49]

## Data Availability

No new data were created or analyzed in this study. Data sharing is not applicable to this study.
